# Effect of Electroacupuncture and Counseling on Sub-Threshold Depression: A Study Protocol for a Multicenter Randomized Controlled Trial

**DOI:** 10.3389/fpsyt.2020.00346

**Published:** 2020-04-28

**Authors:** Xiaotong Wang, Haixiong Lin, Xiumin Jiang, Minna Ma, Dandan Shi, Chun Fan, Yin Shao, Shengwei Wu, Lin Yu, Danian Li, Jun He, Yongjun Chen

**Affiliations:** ^1^South China Research Center for Acupuncture and Moxibustion, Clinical Medical College of Acupuncture, Moxibustion and Rehabilitation, Guangzhou University of Chinese Medicine, Guangzhou, China; ^2^The First School of Clinical Medicine, Guangzhou University of Chinese Medicine, Guangzhou, China; ^3^Rehabilitation Center, Counseling Department, The First Affiliated Hospital of Guangzhou University of Chinese Medicine, Guangzhou, China; ^4^Student Mental Health Counseling Center, Guangzhou University of Chinese Medicine, Guangzhou, China; ^5^Department of Traditional Chinese Medicine, Affiliated Brain Hospital of Guangzhou Medical University, Guangzhou, China; ^6^Guangdong-Hong Kong-Macao Greater Bay Area Center for Brain Science and Brain-Inspired Intelligence, Guangdong Province Key Laboratory of Psychiatric Disorders, Guangzhou, China

**Keywords:** electroacupuncture, counselling, sub-threshold depression, protocol, randomized controlled trial

## Abstract

**Background:**

Sub-threshold depression is common and could impair function, as well as increase the risk of developing major depression. Despite evidence of efficacy for electroacupuncture (EA) and counseling in the treatment of sub-threshold depression, the sample size is insufficient and the level of evidence remains low. This study aims to evaluate the effectiveness of sub-threshold depression treatments by comparing the treatment effects among EA, counseling, and combination therapy, as well as to further study their mechanism.

**Methods:**

This study is a multicenter, randomized, single blind clinical trial that will be conducted in settings at four clinical centers in China. The randomized controlled trial (RCT) will examine the effectiveness of EA intervention, compared with counseling and combination therapy. A total of 138 sub-threshold depression patients (18 to 55 years of age with Beck Depression Inventory (BDI-II) score ≥ 14 points and Hamilton Depression Scale (HAMD-17) score: 7 points ≤ HAMD total score <17 points) will be recruited. The participants will be randomly assigned to receive the above treatments. The interventions will be delivered over a 6-week period (EA: 3 times a week for 6 weeks; 30 min a session. Counseling: once a week for 6 weeks; 50–60 min a session). The primary outcome measure will be the HAMD-17; BDI-II. The secondary outcome measures will be: Self-rating Depression Scale (SDS), Self-rating Anxiety Scale (SAS), and Pittsburgh Sleep Quality Index (PSQI). The assessments will occur at baseline, 2, 4, and 6 weeks and a follow-up period. Recruitment will commence in March 2020 and is anticipated to occur over a 2-year period.

**Discussion:**

This study intends to conduct a multicenter randomized controlled trial to compare the effectiveness among EA, counseling and the combined therapy in the treatment of patients with sub-threshold depression, and to further study the mechanisms of effect.

**Chinese Clinical Trial Registry registration:**

www.chictr.org.cn/, identifier ChiCTR1900028530.

## Introduction

Sub-threshold depression is commonly referred to as “subsyndromal depression” or “minor depression,” and it is mainly characterized by depressive symptoms for at least 2 weeks that fall short of full diagnostic criteria for major depressive disorder (MDD) or dysthymia (1). Epidemiological surveys show that the percentage of 1-year prevalence sub-threshold depression in the general population is 8.4% (2). In China, the incidence of sub-threshold depression among high school students is 22.9%, while college students have reached 36.56% (3, 4). Having consistent depressive symptoms below the threshold criteria is a chronic and disabling condition, with high risks (5, 6) that has a considerable impact on the quality of a patient's life (7). Though sub-threshold falls short of diagnostic criteria, it is as prevalent as more severe depression (8, 9). Additionally, previous studies also indicated that individuals with sub-threshold depression are at a high risk of developing MDD (10–12). Individuals with sub-threshold depression have an odds ratio of more than five for having a first lifetime episode of MDD (13).

Intervening at an early stage could help relieve symptoms and reduce the risk of major depression as well as prevent progression to other undesirable outcomes (8). However, no clinical guidelines or standards for sub-threshold depression have been established. Existing drug interventions have significant side effects (14, 15) and treatment normally causes an economic burden for middle class or low income households, and for the government (16). Currently experts do not recommend standard use of antidepressants as a first-line treatment to those with sub-threshold depression (17, 18), because there is less evidence to prove their effectiveness (19). A meta-analysis found that psychological therapy reduces adulthood depressive symptoms and prevents potential MDD with sub-threshold depression (7). Preventive psychotherapy-based intervention aimed at sub-threshold depression in children and adolescents also shows promising results (12). A previous study found that the psychotherapy intervention has more benefits for alleviating sub-threshold depression symptoms compared to medication management (20). Acupuncture as a major non-drug therapy has been well documented for the treatment of depression (21–23). A cross-sectional study found that depression is the second most common of the most commonly treated acupuncture indications in the United States (24). A single-center controlled trial of sub-threshold depression found that EA, counseling, and EA + counseling could significantly improve HAMD-17 scores, Center for Epidemiologic Depression scale, and WHO Quality of Life-Brief version scores of undergraduates students, and the remission rate is higher than that of the control group (p<0.05) (25). However, the clinical value of this finding is limited because it is a single-center clinical trial with a small sample size. Therefore, a high-quality multi-center RCT with large sample sizes is needed to further confirm this finding, which will provide reliable clinical evidence for the treatment of sub-threshold depression.

Therefore, this study intends to conduct a multicenter randomized controlled trial to compare the effectiveness among EA, counseling and the combined therapy in the treatment of patients with sub-threshold depression, and to further study the mechanism of EA and counseling in the treatment of sub-threshold depression, which will provide more options for the treatment of sub-threshold depression, and provide possible solutions for better efficacy.

## Methods

This protocol was designed according to the Standards for Reporting Interventions in Clinical Trials of Acupuncture (STRICTA) 2010 checklist (**Supplementary Table 1**), CONSORT 2010 checklist with the Non-pharmacological Trials Extension to CONSORT (**Supplementary Table 2**) and registered in the Chinese Clinical Trial Registry (ChiCTR1900028530).

### Trial Objective

We suspect that EA could alleviate the clinical symptoms of patients with sub-threshold depression as well as counseling. If EA is not only the placebo, the combination therapy will have a better effect, and may reduce the risk of conversion from mild to severe, as well as help determine whether patients with sub-threshold depression will need multiple therapies in the future. If acupuncture does no effect, the combination group will have the same clinical effect as the counseling group. Therefore, the objective of this trial is to evaluate the effectiveness of sub-threshold depression treatment by comparing the effects among EA, counseling, and combination therapy, as well as to further study the mechanism of EA and counseling.

### Trial Design

**Figure 1:**
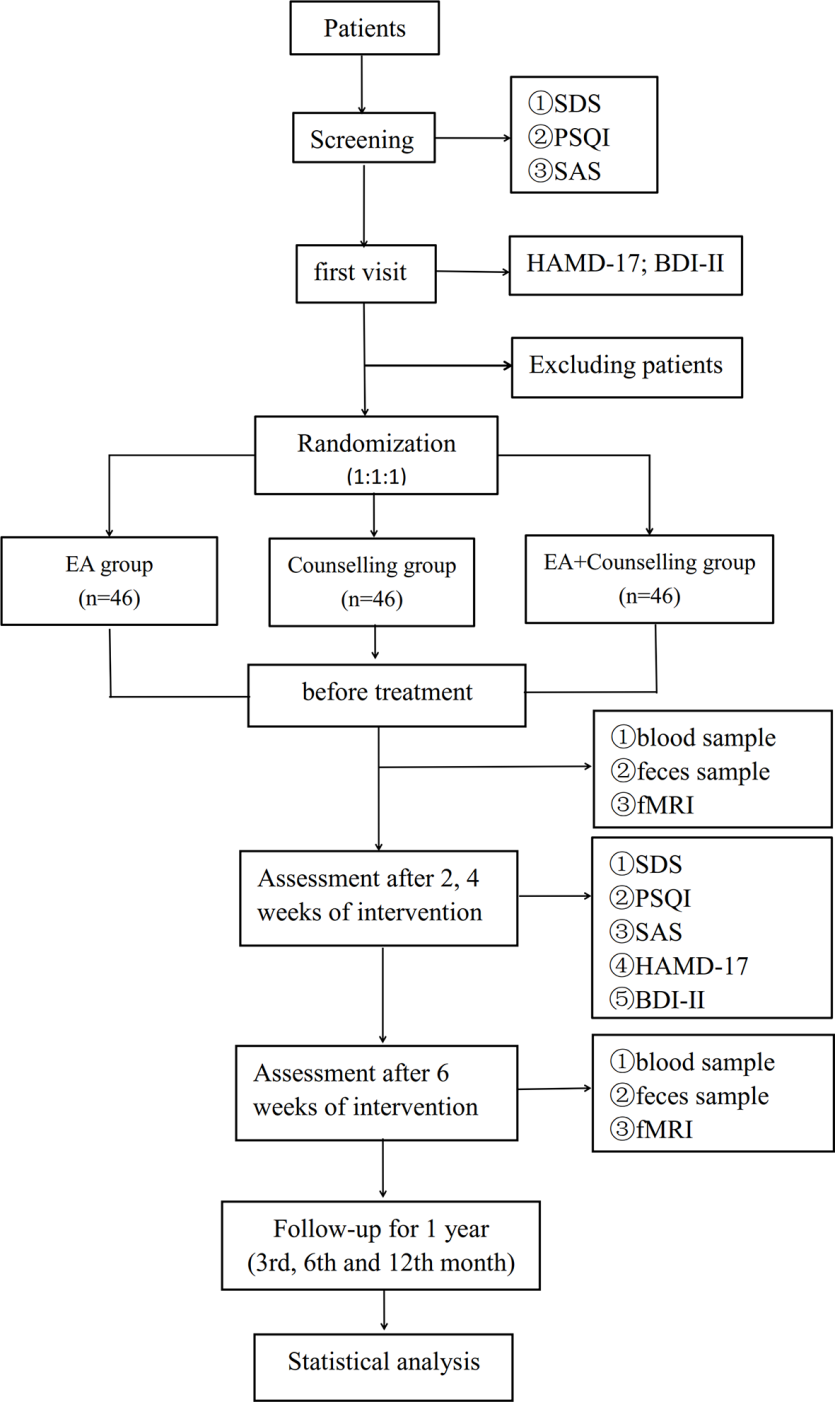
Flow chart of study design.

This study will be a multicenter, randomized, single blind clinical trial with three treatment groups (EA, counseling, and combination therapy). The flow chart in **Figure 1** shows more details regarding the clinical procedures. This study protocol has been approved by the Ethics Committee of the First Affiliated Hospital of Guangzhou University of Chinese Medicine, Affiliated Brain Hospital of Guangzhou Medical University, Guangdong Provincial Hospital of Chinese Medicine, and Guangdong Sanjiu Brain Hospital (NO. ZYYECK YJ [2019] 068).

### Setting

Investigators will conduct the trial in four hospitals in Guangzhou, China. These four hospitals are: The First Affiliated Hospital of Guangzhou University of Chinese Medicine; Affiliated Brain Hospital of Guangzhou Medical University; Guangdong Provincial Hospital of Chinese Medicine; and Guangdong Sanjiu Brain Hospital.

### Blinding

As a single-blinded trial, only outcome evaluators and statistical analysts will be blinded to treatment group. Evaluators and statistical analysts will not be allowed to participate in the treatment process of subjects and will be forbidden from knowing the grouping of subjects. Trial grouping information will be open to subjects, physicians, and data collectors. The analysts will generate a randomized sequence, only known by him or her, to allocate subjects accordingly to ensure the randomization of the grouping. The analyst will be responsible for the preservation and confidentiality of information and data and will prepare a backup of both electronic devices and paperwork. Special personnel will be in charge of those data and documentation. In addition, to ensure strict implementation of the blinded method in the test and reduce the bias of conclusions, physicians (psychologists and acupuncturists), outcome evaluators, data collectors, and statisticians will all be independent of each other and will have no communication during the test.

### Randomization

Independent statisticians will use the block randomization method with SAS version 9.4 (SAS Institute. Inc., Cary, NC) to create a randomization table for each hospital. Randomization numbers will be sealed in opaque, sequentially numbered envelopes, and sent to each participating hospital (26). Random number envelopes will be kept in a double locker. If the participant meets the inclusion criteria and voluntarily signs the informed consent, the clinical research coordinator will send the sealed random number envelopes to the physician in order, who will open the envelope and allocate the qualified participants to the EA group, counseling group, and combined therapy group according to the random number.

### Participants

Participants will be recruited by advertisement and doctor referrals from psychology department clinics. Interested individuals can contact research assistants by email or phone. A series of criteria and prerequisites will be sent to the prospective volunteers for filtering the most eligible subjects. First, registration information and pre-selection query forms with Self-rating Depression Scale (SDS), Pittsburgh Sleep Quality Index (PSQI), and Self-rating Anxiety Scale (SAS) will be sent to registered volunteers. Volunteers will be required to complete the form as a first-round selection prior to scheduling a first visit. This visit will further screen and evaluate a volunteer's eligibility according to inclusion criteria (**Table 1**). Research assistants will collect signed consent forms from volunteers who meet all prerequisites and agree to participate in the trial. Patients will not be included in the study if they meet one of the exclusion criteria (**Table 2**).

**Table 1:** Inclusion criteria.

No	Item	Answer
1	18<age<55	□Yes
		□No
2	Presence of ≥ 1 but < 5 symptoms required for the diagnosis of major depression as detailed in the Diagnostic Statistical Manual of mental disorders (DSM-V), including loss of enjoyment/interest, marked tiredness or reduction of energy, agitation or psychomotor retardation, feelings of worthlessness or guilt, sleep disorders, reduced concentration, loss of appetite and body weight, loss of libido, recurrent suicidal thoughts;	□Yes
		□No
3	symptoms lasting at least 2 weeks	□Yes
		□No
4	BDI-II score≥14 points (24)	□Yes
		□No
5	HAMD-17 score: 7 points ≤ HAMD total score < 17 points (25)	□Yes
		□No

**Table 2:** Exclusion criteria.

No	Item	Answer
1	Patients diagnosed with depression, previous mental illness, or organic mental disorders	□Yes
		□No
2	Patients suffering from serious diseases such as heart, brain, liver, kidney, or hematopoietic system disease	□Yes
		□No
3	Women during pregnancy and lactation or women of childbearing age who have fertility intentions	□Yes
		□No
4	Patients with depressive episodes caused by psychoactive substances and non-addictive substances	□Yes
		□No
5	Patients prescribed other drugs or therapies for treating depression during the trial	□Yes
		□No
6	Individuals addicted to alcohol or other drugs	□Yes
		□No
7	Patients with strong suicidal ideation	□Yes
		□No
8	Patients who are participating in other clinical trials at the same time	□Yes
		□No
9	Patients who have undergone antidepressant treatment within 1 week before the test	□Yes
		□No
10	Skull defects in the acupuncture site, or a surgical scar or skin infection that affects the treatment	□Yes
		□No
11	Patients who are allergic to acupuncture or faint when they are stuck with needles	□Yes
		□No
12	Age older than 55 years or younger than 18 years.	□Yes
		□No

### Elimination and Withdrawal Criteria

1. Misdiagnosis;
2. Those who fail to follow the prescribed treatment or for whom there is incomplete data that would affect the efficacy evaluation and safety evaluation;
3. Subjects with poor compliance, or who use self-administered antidepressants and sleeping pills during the course of treatment, or change the treatment method on their own.

### Observation Criteria That Would Stop the Trial

1. Fail to adhere to the treatment requirement;
2. Cases with serious adverse events that are not suitable for continued treatment;
3. Serious clinical complications that occur in other clinical trials;
4. The progression of disease during treatment.

### Sample Size

Sample size estimation uses a completely random design to compare multiple sample means. Target sample size was estimated using the PASS11 version, 1-β = 0.9, α = 0.05. According to a previous study (27), the mean of HAMD-17 in the psychological counseling group was 5.7, and the standard deviation was 3.13; the mean of HAMD-17 in the EA group was 5.26, and the standard deviation was 3.11; the mean of HAMD-17 in the combined therapy group was 3.4. The standard deviation was 2.97. To reduce the error, the target sample size was slightly enlarged, and a 10% dropout rate was estimated. Therefore, a sample of 46 participants will be the goal for each group in the proposed trial. A total of 138 patients will be aimed for in total.

### Intervention

According to the diagnostic criteria, inclusion criteria, and exclusion criteria, a total of 138 patients with sub-threshold depression will be randomly divided into three groups: EA group, counseling group, and combined therapy group.

### EA Group

**Figure 2:**
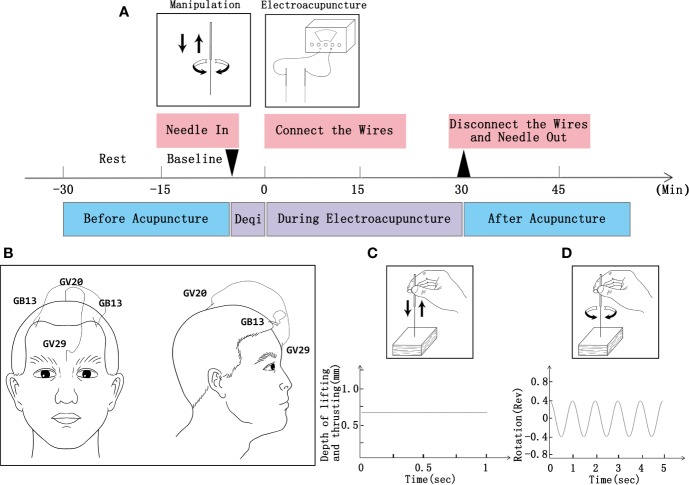
Procedure of EA and operation parameters. **(A)** Schematic protocol of EA manipulation (3 times a week for 6 weeks; 30 min a session). **(B)** Locations of acupoints. **(C)** Operation parameters for lifting and thrusting. **(D)** Operation frequency of twirling and rotation.

Participants will receive 24 sessions of acupuncture treatment (three times a week for 6 weeks; 30 min a session; **Figure 2A**). According to traditional Chinese medicine theory, animal experiments (28, 29), and based on mutual consensus from clinical expert meetings, we will use acupoints GV20, GV29, and bilateral acupoints GB13 (**Figure 2B**) in the treatment. Disposable stainless steel needle insertion will be followed by performed stimulation, such as lifting and thrusting the needle combined with twirling and rotating the needle sheath (as shown in **Figures 2C**, **D**, the operation parameters of depth and frequency will be analyzed by acupuncture manipulation parameter tester [ATP-II; Shanghai Shangyou Medical Equipment Co. Ltd, Shanghai, China]), to produce the sensation known as deqi (sensation of numbness, soreness, radiating, or distention, which is considered to indicate effective needling) (30). One EA wire will be connected to the needle on acupoints GV20 and GV29, and another wire will be connected to the bilateral acupoints GB13. A continuous wave will be provided with a stimulation frequency of 2 Hz (31–33) and an intensity varied from 3 to 5 mA (34) until patients feel comfortable. Acupuncture will be performed by licensed acupuncturists with more than 3 years of clinical experience.

### Counseling Group

Sub-threshold depressed participants will receive individual counseling (once a week for 6 weeks; 50–60 min a session). Counseling will be performed by licensed psychologists with more than 3 years' experience and will be conducted in the psychiatric department of the hospital. Before the trial, the psychologists will receive brief training in counseling and conduct counseling activities based on a treatment manual designed by *Mental Disorders* (35). It mainly includes two aspects: First, help participants analyze their own problems, inform them about the physical harm of psychological defects, and encourage them to overcome these factors. Second, encourage participants to do things of interest to increase excitement and improve emotional state.

### Combination Therapy Group

Participants will receive 24 sessions of EA treatment (three times a week for 6 weeks; 30 min a session) combined with individual counseling (once a week for 6 weeks; 50–60 min a session). Treatment details are the same as the EA and the counseling mentioned above.

Patients will be discouraged from any extra treatment beyond this trial requirement. If this trial does not show an obvious effect after 2 weeks of treatment (HAMD 17 scale score maintained at 7–17 points), or a patient's symptoms have worsened (HAMD 17 scale score > 17 points), we will then consider drug intervention to ensure the safety of the patients. At this point, participants will be forced to stop the trial, and relevant data will be analyzed for intention-to-treat (ITT). Prescription drugs will be assessed under the guidance of a psychiatrist. Such assessment will need to fully consider the physiological characteristics of patients, including age, physical condition, drug tolerance, presence or absence of comorbidities, and prescribing based on guidelines for depression or relevant clinical consensus.

### Safety and Adverse Events

The progress and safety data of the clinical trial will be monitored and managed by an independent Data and Safety Monitoring Committee. The committee will remind participants to report any undesirable and unexpected experiences, whether related to the intervention or not, to the research team at any time during the trial. In addition, the time of occurrence, severity, management, and outcome of these adverse events will need to be recorded on the case report forms. Common adverse events related to EA are fainting during acupuncture, local hematoma, local infection, stucking of needle, broken needle, or bending of needle. The main causes of these adverse events include poor disinfection, improper operation, improper use of needles, and unexpected reactions from patients themselves. The research team will need to explain the work to the patient before starting acupuncture. To achieve proficiency, we will conduct training about acupuncture therapy for acupuncturists. In the case of an adverse event, the researcher, in addition to providing an immediate positive treatment to the patient, will be required to complete an adverse event report and escalate the case to the staff in charge of the research center and the responsible unit for clinical research. All adverse events will be followed up from the reported date until the incident is resolved. According to the WHO Uppsala Monitoring Centre System for Standardized Case Causality Assessment, the research team will evaluate the causality between adverse events and interventions (34).

###  Outcome Measures

The primary outcome measure is the HAMD-17 and BDI-II. Evaluation timelines are set as: before treatment, and the second, fourth, and sixth weeks of the treatment. HAMD-17 and BDI-II are widely used in the diagnosis and outcome evaluation of depression. The range of the HAMD-17 score is 0 to 52, with 25 scores or more indicating severe major depressive disorder, 18–24 scores indicating moderate, 7–17 scores indicating patients may have mild depression, and 0–6 scores indicating normal (36). The range of the BDI-II score is 0 to 63, with 29–63 scores indicating severe major depressive disorder, 20–28 scores indicating moderate, 14–19 scores indicating mild, and 0–13 scores indicating normal. The secondary outcome measures are SDS, SAS, and PSQI. The range of the SDS standard score is 0 to 100, with 72 scores or more indicating severe major depressive disorder, 63–72 scores indicating moderate, 53–62 scores indicating mild, and 0–53 scores indicating normal. The range of the SAS standard score is 0 to 100, with 70 scores or more indicating severe anxiety, 61–70 scores indicating moderate, 50–60 scores indicating mild, and 0–50 scores indicating normal. The range of the PSQI score is 0 to 15, where a lower score indicates better sleep quality. A score of 11–15 indicates poor quality of sleep, and a score of 0–5 indicates good quality of sleep (37). Evaluation time points are as follows: before treatment, and the second, fourth, and sixth weeks of the treatment, and a follow-up period. Participants will be followed up at 3, 6, and 12 months after treatment and will complete the SDS, SAS, and PSQI. In addition, functional MRI (fMRI) is an optional test under the attending physicians' supervision and instruction or depending on the patient's self-willingness. The fMRI, if included, will be performed in the resting state before the treatment and at the sixth week of treatment (38). The detailed procedures and parameters of fMRI are provided in **Supplementary File 1**. Also, the patient's blood samples and feces will be obtained twice before and after the treatment and will be used to build a biological sample bank, which could help with the research protocol moving forward.

### Statistical Analysis

Statistical analysis will be performed by qualified statisticians in a blinded manner using Statistical Product and Service Solutions (SPSS) software (IBM Corporation, Version 21.0). An ITT analysis will be performed. The baseline comparability of the three groups will be assessed on demographic and clinical characteristics, as well as baseline data. Analysis of variance will be used to compare the mean changes between groups from baseline to each time point. The withdrawal of any subjects from clinical trials will be statistically described one by one. The missing data of a withdrawn patient will be randomly filled with the filling method based on the principle of multiple imputations (34). Continuous variables will be represented by mean ± standard deviation. The data before and after treatment in the group will be compared based on a paired *t*-test, and the comparison between groups will be performed using an independent sample *t*-test. When data are not normally distributed, a Wilcoxon rank sum test or Fisher's exact test will be performed. Multiple measurements of the same indicator at different times will use repeated measures data analysis of variance. Categorical variables will be expressed as frequency (%). Two-tailed *p* values < 0.05 will be considered as statistically significant. Safety will be assessed by adverse events and tabulations of adverse drug reactions and descriptive statistics will be performed. Age and intervention assessment are continuous variables, and their correlation will be analyzed using Pearson correlation coefficients. Treatment duration and gender are graded variables, and the spearman correlation coefficient will be used for the correlation analysis of the intervention assessment. To further evaluate the credibility of the blind method. We will evaluate the implementation of the blind method by trial participants' guesses about the allocation of treatment plans, that is, forcing evaluators and statistical analysts to guess the groups to which the subjects are assigned, and then calculate the Blinding Index Scale based on the guess results (39). In order to balance the heterogeneity of patients from different research centers, we will first analyze the differences between the clinical effects of different interventions in each subcenter. If the conclusions of the subcenters are consistent, there will be no central effect. If the conclusions of the sub-centers are inconsistent, a logistic regression analysis will be performed, and the center will be adjusted as a covariate to analyze the results of interventions and clinical assessment (40, 41).

### Quality Control

All types of instruments, equipment, and reagents used for various inspection items in clinical trials will have strict quality standards, to ensure a smooth workflow. Prior to recruitment, every member of the research team, including psychologists, research nurses, and acupuncturists, as well as research assistants in all centers will be required to take part in a training workshop. This requires all personnel to adhere to the research protocol and be familiar with the clinical trial management process. Acupuncturists who perform the treatment will be required to have a certificate as a certified physician issued by the Ministry of Health of the People's Republic of China and have more than 3 years of practical clinical experience. All observation results and abnormal findings in the clinical trial will be carefully verified and recorded on time to ensure the reliability of the data. Research assistants will oversee data collection and constantly check data quality. The various conclusions of the clinical trial must be derived from raw data. To reduce the withdrawal of patients, it will be necessary to streamline the trial procedure, follow-up on time, have good communication with patients, and issue subsidies in a timely manner. As for the dropout rate, whether it occurs during the treatment phase or the follow-up period, the reason should be stated, and the dropout rates should be statistically analyzed.

## Discussion

Depression results in a heavy burden on medical care, severe consequences for society, and huge psychological and health burdens on patients (42). Intervening early could alleviate symptoms, reduce the risk for the development of major depression, prevent progression to other adverse consequences, and reduce the economic burden to the patients and society to a certain extent. In addition, there is a lack of clear diagnostic criteria for sub-threshold depression in ICD-10 and the DSM-V. Thus, based on patient symptoms and rating scale scores, we aim to obtain high-quality research data through a multicenter clinical trial to provide a scientific and reliable basis for future research.

EA has been widely used to treat different psychiatric conditions, including depressive disorder (43). The putative antidepressant actions of EA are believed to involve modulation of hormones, neurotransmitters, and/or cytokines (25). Le et al. also found that EA could regulate the hypothalamus-pituitary-adrenal cortex axis, influence the hippocampus, and affect the dopaminergic and/or serotonergic systems to exert antidepressive activity (44). To date, few studies have been designed and conducted to investigate EA for sub-threshold depression. The goal of this RCT is to collect comparable data as well as to assess the usefulness, advantages, and effectiveness of EA treatment, in comparison to and in combination with counseling for alleviating sub-threshold depression. According to the main treatment principles of regulating the Governor Vessel with acupuncture, we have selected acupoints GV20 and GV29 in GV for EA to improve the curative effect for treating sub-threshold depression. In terms of imageology, EA at acupoint GV20 was previously found to modulate the default pattern network for patients with depression (45). We chose GV20 and GV29, and the bilateral acupoints GB13, because their stimulating area includes the prefrontal cortex. Prefrontal lobe dysfunction is pathophysiologically linked to depression (46). Previous studies confirmed that transcranial direct current stimulation of the prefrontal cortex is a method of treating depression (46, 47). We deduced that the location of our EA could also treat the subthreshold depression by stimulating the dorsolateral prefrontal cortex. The fMRI is a non-invasive test, and it can clearly show the brain area we want to observe (48). It is often used in depression or subthreshold depression studies to determine the efficacy (49, 50). Therefore, if the participants choose to perform fMRI, fMRI will be performed in the resting state before the treatment and at the sixth week of treatment (38).

In terms of clinical outcome assessment, we selected HAMD-17, BDI-II as the primary indicator, and SDS, SAS, and PSQI as the secondary indicators. HAMD-17 was developed by Max Hamilton in 1960 to assess the severity of depressive symptoms (51). BDI-II is a widely used assessment method to measure the presence and severity of depression-related symptoms (52). Therefore, we used HAMD-17 and BDI-II as the primary outcome indicators for assessing the degree and changes of depression in patients. SDS is a short self-assessment scale used to assess the psychological and physical symptoms of depression. It has been widely used for screening purposes and to measure depression in all ages (53). SAS was designed by William WK Zung in 1971 to evaluate the degree of anxiety in patients (54). PSQI is a widely used self-reported sleep questionnaire measure to assess sleep quality, including sleep duration, latency, efficiency, disorders, quality, and daytime dysfunction (55). Previous studies have found that patients with depression are often accompanied by symptoms of anxiety and insomnia (56). Therefore, we used SDS, SAS, and PSQI as secondary outcome indicators.

Some of the key technical issues that need to be addressed in this trial are: (1) in view of the current problems in the treatment of subthreshold depression, this study will explore whether acupuncture could be used as a new non-drug therapy to avoid the deterioration of subthreshold depression and promote the effect of counseling. (2) Determining the value and advantages of acupuncture treatment (effectiveness, onset time, duration of maintenance, side effects, patient compliance, and economic burden). (3) We will establish a biological sample bank before and after acupuncture treatment for patients with subthreshold depression for use in future research.

### Limitation

Our study has some limitations. First of all, in terms of grouping, we cannot achieve double blindness, because acupuncture and counseling belong to different fields, so it is difficult to implement blindness for patients. Second, as all research centers are located in China, it may not be possible to recruit participants from other regions or countries, so the validity of different races needs to be further verified. Thirdly, since the follow-up period is only 12 months, information about long-term prognostic assessment cannot be provided.

## Ethics Statement

This study protocol was approved by the Ethics Committee of the First Affiliated Hospital of Guangzhou University of Chinese Medicine, Affiliated Brain Hospital of Guangzhou Medical University, Guangdong Provincial Hospital of Chinese Medicine, and Guangdong Sanjiu Brain Hospital (NO. ZYYECK YJ [2019] 068). Written informed consent to participate in this study will be provided by the participants.

## Author Contributions

This protocol was first conceived by YC, JH, DL, and LY with critical contributions from the other authors. XW and HL drafted the protocol, designed the picture, and submitted the registration on Chinese Clinical Trial Registry. MM, DS, CF, SW, XJ, and YS revised the manuscript and provide advice. All authors contributed constructive comments on the paper and approved the final protocol.

## Funding

This work was supported, in part, by Science and Technology Program of Guangdong (2018B030334001); National Natural Science Fund of China (81973948); Guangdong Province Universities and Colleges Pearl River Scholar Funded Scheme [2016]; Innovation Team Program of Guangdong Provincial Department of education [grant number 2018KCXTD006]; Grant of Guangdong Province Key Laboratory of Psychiatric Disorders [grant number N201801] and Excellent Doctoral Dissertation Incubation Grant of Guangzhou University of Chinese Medicine (No. GZYXB[2020]18).

## Conflict of Interest

The authors declare that the research was conducted in the absence of any commercial or financial relationships that could be construed as a potential conflict of interest.
