# Supplementary material for: Effect of Electroacupuncture and Counseling on Sub-Threshold Depression: A Study Protocol for a Multicenter Randomized Controlled Trial
Source: Front Psychiatry. 2020 Apr 28;11:346. doi: 10.3389/fpsyt.2020.00346 (PMC7198880; doi:10.3389/fpsyt.2020.00346)
Supplement: Supplementary file 1 [file DataSheet_1.doc]

**Supplementary File 1. Scanning devices and parameters of functional MRI (fMRI)**

Brain function data in resting state of all subjects were collected by a German SIMENS 3.0t Prisma magnetic resonance imaging (MRI) machine. During the MRI examination, subjects lie on the scan table, remain awake, close their eyes to avoid systemic thinking. Subjects were instructed to wear earphones to reduce noise interference, and their heads were fixed with foam pads to reduce head movement. MRI signals are received using a standard 64 channel head coil. Prior to fMRI testing, routine whole-brain axial T1WI, T2WI and FLAIR sequence scans were performed to screen for brain disorders. Gradient echo sequences-echo planar imaging (GRE-SS-EPI) sequences were used for the collection of resting-state BOLD-fMRI data. TR=500ms, TE=30ms, layer thickness 3mm, layer spacing 1mm, field of view (FOV) =220×220mm, matrix 64×64, turning Angle 90°, and 185 time points were collected. Collect MRI slices sequentially from the base of the skull to the top of the skull. The scanning parameters of the 3D-T1 were as follows: TR=6.9ms,TE=1.5ms, flip Angle (FA)=12°, layer thickness =1mm, layer number 188, matrix =256×256, FOV=256×256mm, and the whole brain scan was conducted parallel to the median sagittal plane.
