# Supplementary material for: Effect of Electroacupuncture and Counseling on Sub-Threshold Depression: A Study Protocol for a Multicenter Randomized Controlled Trial
Source: Front Psychiatry. 2020 Apr 28;11:346. doi: 10.3389/fpsyt.2020.00346 (PMC7198880; doi:10.3389/fpsyt.2020.00346)
Supplement: Supplementary file 2 [file Table_1.doc]

**Supplementary Table 1: STRICTA 2010 checklist of information to include when reporting interventions in a clinical trial of acupuncture**

| **Item** | **Detail** | **Page** |
| --- | --- | --- |
| 1. Acupuncture rationale | (a) Style of acupuncture (eg, TCM, Japanese, Korean, Western medical, Five Element, ear acupuncture, etc) | **P1** |
|  | (b) Reasoning for treatment provided, based on historical context, literature sources, and consensus methods, with references where appropriate | **P2** |
|  | (c) Extent to which treatment was varied | **P2** |
| 2. Details of needling | (a) Number of needle insertions per subject per session (mean and range where relevant) | **P4-5** |
|  | (b) Names (or location if no standard name) of points used (uni/bilateral) | **P4-5** |
|  | (c) Depth of insertion, based on a specified unit of measurement, or on a particular tissue level | **P4-5** |
|  | (d) Response sought (eg, de qi or muscle twitch response) | **P4-5** |
|  | (e) Needle stimulation (eg, manual, electrical) | **P4-5** |
|  | (f) Needle retention time | **P4-5** |
|  | (g) Needle type (diameter, length, and manufacturer or material) | **P4-5** |
| 3. Treatment regimen | (a) Number of treatment sessions | **P4-5** |
|  | (b) Frequency and duration of treatment sessions | **P4-5** |
| 4. Other components of treatment | (a) Details of other interventions administered to the acupuncture group (eg, moxibustion, cupping, herbs, exercises, lifestyle advice) | **P4-5** |
|  | (b) Setting and context of treatment, including instructions to practitioners, and information and explanations to patients | **P7** |
| 5.Practitioner background | Description of participating acupuncturists (qualification or professional affiliation, years in acupuncture practice, other relevant experience) | **P5** |
| 6. Control or comparator interventions | (a) Rationale for the control or comparator in the context of the research question, with sources that justify this choice | **P2** |
|  | (b) Precise description of the control or comparator. If sham acupuncture or any other type of acupuncture-like control is used, provide details as for Items 1 to 3 above. | **P4-5** |

Note: This checklist, which should be read in conjunction with the explanations of the STRICTA items provided in the main text, is designed to replace CONSORT 2010’s item 5 when reporting an acupuncture trial.
