# Supplementary material for: Effect of Electroacupuncture and Counseling on Sub-Threshold Depression: A Study Protocol for a Multicenter Randomized Controlled Trial
Source: Front Psychiatry. 2020 Apr 28;11:346. doi: 10.3389/fpsyt.2020.00346 (PMC7198880; doi:10.3389/fpsyt.2020.00346)
Supplement: Supplementary file 3 [file Table_2.doc]

**Supplementary Table 2: CONSORT 2010 checklist with the Non-pharmacological Trials Extension to CONSORT (with STRICTA 2010 extending CONSORT Item 5 for acupuncture trials)**

| **Section/Topic** | **Item #** | **CONSORT 2010 Statement*: Checklist item. Describe:** | **Additional items from the Non-pharmacological Trials Extension to CONSORT. Add:** | **Page** |
| --- | --- | --- | --- | --- |
| ***TITLE AND ABSTRACT*** | | | | |
|  | 1.a | Identification as a randomized trial in the title | In the abstract, description of the experimental treatment, comparator, care providers, centres and blinding status. | P1 |
|  | 1.b | Structured summary of trial design, methods, results, and conclusions; for specific guidance see CONSORT for Abstracts |  | P2 |
| ***INTRODUCTION*** | | | | |
| Background and objectives | 2.a | Scientific background and explanation of rationale |  | P2 |
|  | 2.b | Specific objectives or hypotheses |  | P2 |
| **METHODS** | | | | |
| Trial design | 3.a | Description of trial design (e.g., parallel, factorial) including allocation ratio |  | P3 |
|  | 3.b | Important changes to methods after trial commencement (e.g. eligibility criteria), with reasons |  | P3 |
| Participants | 4.a | Eligibility criteria for participants | When applicable, eligibility criteria for centers and those performing the interventions. | P3 |
|  | 4.b | Settings and locations where the data were collected |  | P3 |
| Interventions | 5 | The interventions for each group with sufficient details to allow replication, including how and when they were actually administered | Precise details of both the experimental treatment and comparator - see Table 1 for details | P3 |
| Outcomes | 6.a | Completely defined pre-specified primary and secondary outcome measures, including how and when they were assessed |  | P6 |
|  | 6.b | Any changes to trial outcomes after the trial commenced with reasons |  | P6 |
| Sample size | 7.a | How sample size was determined | When applicable, details of whether and how the clustering by care providers or centers was addressed. | P3 |
|  | 7.b | When applicable, explanation of any interim analyses and stopping guidelines |  | P3 |
| Randomization |  | | | |
| *Sequence generation* | 8.a | Method used to generate the random allocation sequence | When applicable, how care providers were allocated to each trial group. | P3 |
|  | 8.b | Type of randomization; details of any restriction (e.g., blocking and block size) |  | P3 |
| *Allocation concealment* | 9 | Mechanism used to implement the random allocation sequence (e.g., sequentially numbered containers), describing any steps taken to conceal the sequence until interventions were assigned |  | P3 |
| *Implementation* | 10 | Who generated the random allocation sequence, who enrolled participants, and who assigned participants to interventions |  | P3 |
| Blinding | 11.a | If done, who was blinded after assignment to interventions (e.g. participants, care providers, those assessing outcomes) and how | Whether or not those administering co-interventions were blinded to group assignment. If blinded, method of blinding and description of the similarity of interventions. | P3 |
|  | 11.b | If relevant, description of the similarity of interventions |  | P3 |
| Statistical methods | 12.a | Statistical methods used to compare groups for primary and secondary outcomes | When applicable, details of whether and how the clustering by care providers or centers was addressed. | P7 |
|  | 12.b | Methods for additional analyses, such as subgroup analyses and adjusted analyses |  | P7 |
| **RESULTS** | | | | |
| Participant flow (A diagram is strongly recommended) | 13.a | For each group, the numbers of participants who were randomly assigned, received intended treatment, and were analyzed for the primary outcome | The number of care providers or centers performing the intervention in each group and the number of patients treated by each care provider or in each center. | P4 |
|  | 13.b | For each group, losses and exclusions after randomization, together with reasons |  | P4 |
| Implementation of intervention |  |  | Details of the experimental treatment and comparator as they were implemented. | P3 |
| Recruitment | 14.a | Dates defining the periods of recruitment and follow-up |  | P3 |
|  | 14.b | Why the trial ended or was stopped |  | P3, 7 |
| Baseline data | 15 | A table showing baseline demographic and clinical characteristics for each group | When applicable, a description of care providers (case volume, qualification, expertise, etc.) and centers (volume) in each group. | P7 |
| Numbers analyzed | 16 | For each group, number of participants (denominator) included in each analysis and whether the analysis was by original assigned groups |  | P7 |
| Outcomes and estimation | 17.a | For each primary and secondary outcome, results for each group, and the estimated effect size and its precision (e.g., 95% confidence interval) |  | P6-7 |
|  | 17.b | For binary outcomes, presentation of both absolute and relative effect sizes is recommended |  | P6-7 |
| Ancillary analyses | 18 | Results of any other analyses performed, including subgroup analyses and adjusted analyses, distinguishing pre-specified from exploratory |  | P7 |
| Harms | 19 | All important harms or unintended effects in each group; for specific guidance see CONSORT for Harms [60] |  | P6 |
| **DISCUSSION** | | | | |
| Limitations | 20 | Trial limitations, addressing sources of potential bias, imprecision, and, if relevant, multiplicity of analyses |  | P7 |
| Generalizability | 21 | Generalizability (external validity, applicability) of the trial findings | Generalizability (external validity) of the trial findings according to the intervention, comparators, patients and care providers and centers involved in the trial. | P6-7 |
| Interpretation | 22 | Interpretation consistent with results, balancing benefits and harms, and considering other relevant evidence | In addition, take into account the choice of the comparator, lack of or partial blinding, unequal expertise of care providers or centers in each group. | P6-7 |
| ***Other Information*** | | | | |
| Registration | 23 | Registration number and name of trial registry |  | P2 |
| Protocol | 24 | Where the full trial protocol can be accessed, if available |  | P2 |
| Funding | 25 | Sources of funding and other support (e.g., supply of drugs); role of funders |  | P8 |

* We strongly recommend reading this Statement in conjunction with the CONSORT 2010 explanation and elaboration for important clarifications on all the items. If relevant, we also recommend reading CONSORT extensions for cluster randomized trials, noninferiority and equivalence trials, herbal interventions, and pragmatic trials. Moreover, additional extensions are forthcoming. For those and also for up-to-date references relevant to this checklist, see [http://www.consort-statement.org.](http://www.consort-statement.org./)
